# Metropolitan Hospital Sunday Fund

**Published:** 1903-05-16

**Authors:** 


### METROPOLITAN HOSPITAL SUNDAY FUND.

### THEIR MAJESTIES' FORTHCOMING VISIT TO ST. PAUL'S CATHEDRAL.

### MR. GEORGE HERRING GIVES £25,000.

A meeting of the Council of the Metropolitan Hospital Sunday Fund was held at the Mansion House on Tuesday, 12th inst., the Lord Mayor (Sir Marcus Samuel) in the chair.

At the opening of the proceedings Mr. Carr-Gomm moved, and Mr. Hampton Hale seconded, and the Council adopted, a vote of regret on the death, and sympathy with the family, of the Rev. Prebendary Kitto, who had been a member of the Council since its inception.

The Lord Mayor said they had met to consider the best means of furthering the interests of the Hospital Sunday Fund. He had to announce that, except at St. Paul's Cathedral, where, by the King's desire, a special service would be held on June 7th, at which he and her Majesty the Queen would be present, the collections this year for the Fund would be held on Sunday, June 14th. He was also empowered to state that, following on his generous gifts, Mr. George Herring had promised this year to give £25,000, provided £100,000 were raised in the various places of public worship. It would be the duty and pleasure of the Lady Mayoress and himself to attend the service on June 7th, and it would give him the greatest pleasure to receive contributions from all who desired to assist in making a success of this historic occasion, the first on which the Sovereign would attend for the express purpose of lending his countenance to the objects of that Fund; and in order to encourage enthusiastic effort he wished to point out to all interested in the welfare of the hospitals, and to emphasise the fact that they were within measurable distance of attaining their object. The whole deficiency of the London hospitals, provided they kept within the control of the committees in the matter of expenditure, only amounted to £250,000. If the Sunday Fund could raise £100,000, King Edward's Fund £100,000, and the Saturday Fund £50,000, the hospitals would be put in a position of solvency, and it would be seen that there was not the slightest cause for rate assistance. It was now beginning to be realised that the great banking and insurance companies and limited companies generally were entitled to contribute to hospital funds; and since the discovery of this fortunate loophole some of the leading business institutions of the City had so contributed, notably, to the special appeal he himself was making on behalf of the London Hospital. If they would make up their minds to contribute liberally to the central funds they would be spared having to investigate the claims of individual appeals, and they would help to secure efficiency and economy of administration, since hospitals would have no chance of success unless they had the fiat of one or other of these councils, or preferably of a joint authority from all three. As to the feasibility of the Sunday Fund obtaining £100,000 he would point out that last year they got £62,000, so they had only £38,000 increase to make up. He would like to point out that though they had liberal support from the Churches there were a large number of people who did not attend church —"week-enders" they were called—who took rest and recreation in their own way; but that should not free them from the liability that properly devolved upon every citizen, and he thought it would be a good thing if means could be found to bring these gentlemen into the fold and obtain contributions from them to the Fund. He himself had written, not only to the clergymen and ministers of all denominations, but he had sent some 5,000 letters to people living outside London. It should be remembered that no less than 25 per cent. of the patients of the London hospitals came from outlying districts; and as these people did not by reason of the London hospitals have to contribute to hospitals of their own they should recognise their obligation to help such Funds as that. They had just had a conference on the subject when Canon Fleming and Prebendary Ridgeway had been good enough to explain the lines on which they worked so successfully, and pamphlets were being circulated containing their advice. A great meeting was to be held there on June 8th, when the Archbishop of Canterbury, Sir Frederick Treves, and Sir Francis Powell would speak. He hoped for a record collection at St. Paul's on June 7th; and the fact that the King and Queen were coming should ensure it, for they were very loyal in the City; and if they had a record collection it could not fail to wake up the other Churches, and he should hope to see the amount supplemented by a second collection on the 14th. If that were so, it would be one of the proudest memorials of his mayoralty.

Sir Edmund Hay Currie then read the text of Mr. George Herring's offer, which was as follows:—

> I propose to make the same offer as last year to the Council of the Hospital Sunday Fund, namely, I will either send a cheque for £10,000 or add one quarter to the amount collected in all places of public worship. But you are showing such increased energy this year that I must limit the amount to £100,000, so that if the collection exceeds that sum (which I sincerly hope may be the case) I only give £25,000.
>
> Kindly place this letter before the Council and let me know the result.

Sir Henry Burdett said they were indebted to the Lord Mayor for the clear and concise way in which he had presented the case. It was wrong for any hospital or group of hospitals to encourage the idea that the voluntary system was in peril. So far was that from being the case that for the last 25 years the actual margin between expenditure and revenue had gone on growing narrower. When they looked at the figures in the concrete as the Lord Mayor had presented them it was clear that their part of the task was to provide £100,000. They had only £38,000 to make up to reach that figure, and of that sum more than one-third was offered as a gift by Mr. George Herring. Expressed in figures it amounted to this, that if the Metropolitan Hospital Sunday Fund contributed an extra £24,000, he would give an extra £13,425 in addition to the £11,575 he gave last year. That task ought not to be a difficult one. If, as he hoped, the statement were true—and coming from such a source no doubt it was—that the large banking, insurance, and other limited liability companies had at last come to the decision that as they had the power they ought to contribute according to their profits, the task of providing funds for the hospitals would be practically completed. The splendid munificence of Mr. George Herring was a striking example of how the cause of suffering appealed to the man of business—an example which would, he hoped, be imitated in his degree by every citizen.

The Archdeacon of London supported the appeal. He had received a letter from another Mr. Herring—Mr. William Herring—in reply to a request to become a steward in St. Paul's on the occasion of the King's visit. Their secretary had asked a large number of people to become stewards, and in response to that Mr. Herring wrote that his name might be put down for £1,000. For the Festival of the Sons of the Clergy they had a large number of stewards who were expected to pay for the first year 30 guineas and for subsequent years 20 guineas, and he hoped every member of the Council would do his best to secure the services of stewards for June 7th. The duties were not very serious—merely to take charge of a section of the seating—and they ought to do at least as well as the Sons of the Clergy.

The Bishop of Stepney said he should be the Canon in residence on the 7th. He had seen Lord Knollys, and he learned that it was the King's wish that it should be a personal and private service, and that by his presence the support given to that Fund by the City of London should be largely increased. It was the business of the Council to see that the interest and wealth latent in the City should come forward on that occasion. Their plans were at present imperfect, but he thought that invitations for the seats the Dean and Chapter would reserve should be sent by the Council in conjunction with the Lord Mayor, who had the best means of knowing the City, so that those invited might be likely to enter into the spirit of the matter and give largely to the collection.

Prebendary Ridgeway was anxious that it should be made clear that they did not want to draw away from other congregations for this special service at St. Paul's, but by it to increase the interest in the general collection on the following Sunday.

Sir Edmund Currie said the people they wanted to get at were those who did not contribute at other churches. It had been said that six-sevenths of the people in this country did not go to church, and it was the "week-enders" they wanted more particularly on this occasion.

Dr. Glover proposed a vote of thanks to the Lord Mayor, and referred to the danger of robbing Peter to pay Paul. They certainly did not want the collections at individual churches to suffer because prominent members had already been bled for the central fund.

Archdeacon Sinclair seconded the motion, and assured the Council that their intention was to fill the Cathedral very largely with people connected with the City of London, and he sincerely hoped nothing would be done to interfere with individual collections on the 14th.

The Lord Mayor, in reply, said there was always a little friendly rivalry between successive Lord Mayors in these matters, and the presence of Sir Joseph Dimsdale, who was responsible for a record collection in his year of office, induced him to say that he should be glad if he could beat him—and so he was sure would he.

Sir Joseph Dimsdale said no one in the room wished it more than he. He offered his congratulations to the Dean and Chapter of St. Paul's on its development from its shell. It was the one church where in the past the collections were beneath contempt. Last year, however, they had moved in the matter, and had brought the collection up to a decent figure. He hoped the authorities would now be inspired with an enthusiasm which in the past had been distinctly wanting.

The meeting then separated.

